# Supplementary material for: Competitive binding of STATs to receptor phospho-Tyr motifs accounts for altered cytokine responses
Source: eLife. 2021 Apr 19;10:e66014. doi: 10.7554/eLife.66014 (PMC8099432; doi:10.7554/eLife.66014)
Supplement: Supplementary file 1. [file elife-66014-supp1.docx]

**Supplementary material 1**

Equations (1) – (22) are the ordinary differential equations (ODEs) describing the HypIL-6 signalling pathway, under the assumption of mass action kinetics.

| $\frac{d\left[ R_{1} \right]}{dt}=-r_{1,6}^{+}\left[ R_{1} \right]\left[ L_{6} \right]+r_{1,6}^{-}\left[ C_{1} \right]-\beta_{6}\left[ R_{1} \right]-\gamma_{6}\left( \left[ pS_{1} \right]+\left[ pS_{3} \right] \right)\left[ R_{1} \right]$ | (1) |
| --- | --- |
| $\frac{d\left[ L_{6} \right]}{dt}=-r_{1,6}^{+}\left[ R_{1} \right]\left[ L_{6} \right]+r_{1,6}^{-}\left[ C_{1} \right]$ | (2) |
| $\frac{d\left[ C_{1} \right]}{dt}=r_{1,6}^{+}\left[ R_{1} \right]\left[ L_{6} \right]-r_{1,6}^{-}\left[ C_{1} \right]-2r_{2,6}^{+}\left[ C_{1} \right]^{2}+2r_{2,6}^{-}\left[ D_{6} \right]-\beta_{6}\left[ C_{1} \right]-\gamma_{6}\left( \left[ pS_{1} \right]+\left[ pS_{3} \right] \right)\left[ C_{1} \right]$ | (3) |
| $\frac{d\left[ D_{6} \right]}{dt}=r_{2,6}^{+}\left[ C_{1} \right]^{2}-r_{2,6}^{-}\left[ D_{6} \right]-2k_{1a}^{+}\left[ D_{6} \right]\left[ S_{1} \right]+k_{1a}^{-}\left( \left[ D_{6}\cdot S_{1} \right]+\left[ D_{6}\cdot pS_{1} \right] \right)-2k_{3a}^{+}\left[ D_{6} \right]\left[ S_{3} \right]+k_{3a}^{-}\left( \left[ D_{6}\cdot S_{3} \right]+\left[ D_{6}\cdot pS_{3} \right] \right)-\beta_{6}\left[ D_{6} \right]-\gamma_{6}\left( \left[ pS_{1} \right]+\left[ pS_{3} \right] \right)\left[ D_{6} \right]$ | (4) |
| $\frac{d\left[ S_{1} \right]}{dt}=-k_{1a}^{+}\left[ S_{1} \right]\left( 2\left[ D_{6} \right]+\left[ D_{6}\cdot S_{1} \right]+\left[ D_{6}\cdot S_{3} \right]+\left[ D_{6}\cdot pS_{1} \right]+\left[ D_{6}\cdot pS_{3} \right] \right)+k_{1a}^{-}\left( \left[ D_{6}\cdot S_{1} \right]+2\left[ S_{1}\cdot D_{6}\cdot S_{1} \right]+\left[ S_{1}\cdot D_{6}\cdot S_{3} \right]+\left[ S_{1}\cdot D_{6}\cdot pS_{1} \right]+\left[ S_{1}\cdot D_{6}\cdot pS_{3} \right] \right)+d_{1}\left[ pS_{1} \right]$ | (5) |
| $\frac{d\left[ S_{3} \right]}{dt}=-k_{3a}^{+}\left[ S_{3} \right]\left( 2\left[ D_{6} \right]+\left[ D_{6}\cdot S_{3} \right]+\left[ D_{6}\cdot S_{1} \right]+\left[ D_{6}\cdot pS_{3} \right]+\left[ D_{6}\cdot pS_{1} \right] \right)+k_{3a}^{-}\left( \left[ D_{6}\cdot S_{3} \right]+2\left[ S_{3}\cdot D_{6}\cdot S_{3} \right]+\left[ S_{1}\cdot D_{6}\cdot S_{3} \right]+\left[ S_{3}\cdot D_{6}\cdot pS_{3} \right]+\left[ pS_{1}\cdot D_{6}\cdot S_{3} \right] \right)+d_{3}\left[ pS_{3} \right]$ | (6) |
| $\frac{d\left[ D_{6}\cdot S_{1} \right]}{dt}=2k_{1a}^{+}\left[ S_{1} \right]\left[ D_{6} \right]-k_{1a}^{-}\left[ D_{6}\cdot S_{1} \right]-k_{1a}^{+}\left[ D_{6}\cdot S_{1} \right]\left[ S_{1} \right]+2k_{1a}^{-}\left[ S_{1}\cdot D_{6}\cdot S_{1} \right]-k_{3a}^{+}\left[ D_{6}\cdot S_{1} \right]\left[ S_{3} \right]+k_{3a}^{-}\left[ S_{1}\cdot D_{6}\cdot S_{3} \right]-q\left[ D_{6}\cdot S_{1} \right]+k_{1a}^{-}\left[ S_{1}\cdot D_{6}\cdot pS_{1} \right]+k_{3a}^{-}\left[ S_{1}\cdot D_{6}\cdot pS_{3} \right]-\beta_{6}\left[ D_{6}\cdot S_{1} \right]-\gamma_{6}\left( \left[ pS_{1} \right]+\left[ pS_{3} \right] \right)\left[ D_{6}\cdot S_{1} \right]$ | (7) |
| $\frac{d\left[ D_{6}\cdot S_{3} \right]}{dt}=2k_{3a}^{+}\left[ S_{3} \right]\left[ D_{6} \right]-k_{3a}^{-}\left[ D_{6}\cdot S_{3} \right]-k_{3a}^{+}\left[ D_{6}\cdot S_{3} \right]\left[ S_{3} \right]+2k_{3a}^{-}\left[ S_{3}\cdot D_{6}\cdot S_{3} \right]-k_{1a}^{+}\left[ D_{6}\cdot S_{3} \right]\left[ S_{1} \right]+k_{1a}^{-}\left[ S_{1}\cdot D_{6}\cdot S_{3} \right]-q\left[ D_{6}\cdot S_{3} \right]+k_{1a}^{-}\left[ pS_{1}\cdot D_{6}\cdot S_{3} \right]+k_{3a}^{-}\left[ S_{3}\cdot D_{6}\cdot pS_{3} \right]-\beta_{6}\left[ D_{6}\cdot S_{3} \right]-\gamma_{6}\left( \left[ pS_{1} \right]+\left[ pS_{3} \right] \right)\left[ D_{6}\cdot S_{3} \right]$ | (8) |
| $\frac{d\left[ D_{6}\cdot pS_{1} \right]}{dt}=-k_{1a}^{+}\left[ S_{1} \right]\left[ D_{6}\cdot pS_{1} \right]+k_{1a}^{-}\left[ S_{1}\cdot D_{6}\cdot pS_{1} \right]-k_{3a}^{+}\left[ S_{3} \right]\left[ D_{6}\cdot pS_{1} \right]+k_{3a}^{-}\left[ pS_{1}\cdot D_{6}\cdot S_{3} \right]+q\left[ D_{6}\cdot S_{1} \right]-k_{1a}^{-}\left[ D_{6}\cdot pS_{1} \right]+2k_{1a}^{-}\left[ pS_{1}\cdot D_{6}\cdot pS_{1} \right]+k_{3a}^{-}\left[ pS_{1}\cdot D_{6}\cdot pS_{3} \right]-\beta_{6}\left[ D_{6}\cdot pS_{1} \right]-\gamma_{6}\left( \left[ pS_{1} \right]+\left[ pS_{3} \right] \right)\left[ D_{6}\cdot pS_{1} \right]$ | (9) |
| $\frac{d\left[ D_{6}\cdot pS_{3} \right]}{dt}=-k_{3a}^{+}\left[ S_{3} \right]\left[ D_{6}\cdot pS_{3} \right]+k_{3a}^{-}\left[ S_{3}\cdot D_{6}\cdot pS_{3} \right]-k_{1a}^{+}\left[ S_{1} \right]\left[ D_{6}\cdot pS_{3} \right]+k_{1a}^{-}\left[ S_{1}\cdot D_{6}\cdot pS_{3} \right]+q\left[ D_{6}\cdot S_{3} \right]-k_{3a}^{-}\left[ D_{6}\cdot pS_{3} \right]+2k_{3a}^{-}\left[ pS_{3}\cdot D_{6}\cdot pS_{3} \right]+k_{1a}^{-}\left[ pS_{1}\cdot D_{6}\cdot pS_{3} \right]-\beta_{6}\left[ D_{6}\cdot pS_{3} \right]-\gamma_{6}\left( \left[ pS_{1} \right]+\left[ pS_{3} \right] \right)\left[ D_{6}\cdot pS_{3} \right]$ | (10) |
| $\frac{d\left[ S_{1}\cdot D_{6}\cdot S_{1} \right]}{dt}=k_{1a}^{+}\left[ S_{1} \right]\left[ D_{6}\cdot S_{1} \right]-2k_{1a}^{-}\left[ S_{1}\cdot D_{6}\cdot S_{1} \right]-2q\left[ S_{1}\cdot D_{6}\cdot S_{1} \right]-\beta_{6}\left[ S_{1}\cdot D_{6}\cdot S_{1} \right]-\gamma_{6}\left( \left[ pS_{1} \right]+\left[ pS_{3} \right] \right)\left[ S_{1}\cdot D_{6}\cdot S_{1} \right]$ | (11) |
| $\frac{d\left[ S_{3}\cdot D_{6}\cdot S_{3} \right]}{dt}=k_{3a}^{+}\left[ S_{3} \right]\left[ D_{6}\cdot S_{3} \right]-2k_{3a}^{-}\left[ S_{3}\cdot D_{6}\cdot S_{3} \right]-2q\left[ S_{3}\cdot D_{6}\cdot S_{3} \right]-\beta_{6}\left[ S_{3}\cdot D_{6}\cdot S_{3} \right]-\gamma_{6}\left( \left[ pS_{1} \right]+\left[ pS_{3} \right] \right)\left[ S_{3}\cdot D_{6}\cdot S_{3} \right]$ | (12) |
| $\frac{d\left[ pS_{1}\cdot D_{6}\cdot S_{1} \right]}{dt}=k_{1a}^{+}\left[ pS_{1}\cdot D_{6} \right]\left[ S_{1} \right]-2k_{1a}^{-}\left[ pS_{1}\cdot D_{6}\cdot S_{1} \right]$  $+2q\left[ S_{1}\cdot D_{6}\cdot S_{1} \right]-q\left[ pS_{1}\cdot D_{6}\cdot S_{1} \right]-\beta_{6}\left[ pS_{1}\cdot D_{6}\cdot S_{1} \right]$  $-\gamma_{6}\left( \left[ pS_{1} \right]+\left[ pS_{3} \right] \right)\left[ pS_{1}\cdot D_{6}\cdot S_{1} \right]$ | (13) |
| $\frac{d\left[ pS_{3}\cdot D_{6}\cdot S_{3} \right]}{dt}=k_{3a}^{+}\left[ pS_{3}\cdot D_{6} \right]\left[ S_{3} \right]-2k_{3a}^{-}\left[ pS_{3}\cdot D_{6}\cdot S_{3} \right]+2q\left[ S_{3}\cdot D_{6}\cdot S_{3} \right]-q\left[ pS_{3}\cdot D_{6}\cdot S_{3} \right]-\beta_{6}\left[ pS_{3}\cdot D_{6}\cdot S_{3} \right]-\gamma_{6}\left( \left[ pS_{1} \right]+\left[ pS_{3} \right] \right)\left[ pS_{3}\cdot D_{6}\cdot S_{3} \right]$ | (14) |
| $\frac{d\left[ pS_{1}\cdot D_{6}\cdot pS_{1} \right]}{dt}=q\left[ pS_{1}\cdot D_{6}\cdot S_{1} \right]-2k_{1a}^{-}\left[ pS_{1}\cdot D_{6}\cdot pS_{1} \right]$  $-\beta_{6}\left[ pS_{1}\cdot D_{6}\cdot pS_{1} \right]-\gamma_{6}\left( \left[ pS_{1} \right]+\left[ pS_{3} \right] \right)\left[ pS_{1}\cdot D_{6}\cdot pS_{1} \right]$ | (15) |
| $\frac{d\left[ pS_{3}\cdot D_{6}\cdot pS_{3} \right]}{dt}=q\left[ pS_{3}\cdot D_{6}\cdot S_{3} \right]-2k_{3a}^{-}\left[ pS_{3}\cdot D_{6}\cdot pS_{3} \right]$  $-\beta_{6}\left[ pS_{3}\cdot D_{6}\cdot pS_{3} \right]-\gamma_{6}\left( \left[ pS_{1} \right]+\left[ pS_{3} \right] \right)\left[ pS_{3}\cdot D_{6}\cdot pS_{3} \right]$ | (16) |
| $\frac{d\left[ S_{1}\cdot D_{6}\cdot S_{3} \right]}{dt}=k_{1a}^{+}\left[ S_{1} \right]\left[ D_{6}\cdot S_{3} \right]-k_{1a}^{-}\left[ S_{1}\cdot D_{6}\cdot S_{3} \right]+k_{3a}^{+}\left[ S_{1}\cdot D_{6} \right]\left[ S_{3} \right]-k_{3a}^{-}\left[ S_{1}\cdot D_{6}\cdot S_{3} \right]-2q\left[ S_{1}\cdot D_{6}\cdot S_{3} \right]-\beta_{6}\left[ S_{1}\cdot D_{6}\cdot S_{3} \right]-\gamma_{6}\left( \left[ pS_{1} \right]+\left[ pS_{3} \right] \right)\left[ S_{1}\cdot D_{6}\cdot S_{3} \right]$ | (17) |
| $\frac{d\left[ pS_{1}\cdot D_{6}\cdot S_{3} \right]}{dt}=q\left[ S_{1}\cdot D_{6}\cdot S_{3} \right]+k_{3a}^{+}\left[ pS_{1}\cdot D_{6} \right]\left[ S_{3} \right]$  $-k_{3a}^{-}\left[ pS_{1}\cdot D_{6}\cdot S_{3} \right]-q\left[ pS_{1}\cdot D_{6}\cdot S_{3} \right]-k_{1a}^{-}\left[ pS_{1}\cdot D_{6}\cdot S_{3} \right]$  $-\beta_{6}\left[ pS_{1}\cdot D_{6}\cdot S_{3} \right]-\gamma_{6}\left( \left[ pS_{1} \right]+\left[ pS_{3} \right] \right)\left[ pS_{1}\cdot D_{6}\cdot S_{3} \right]$ | (18) |
| $\frac{d\left[ S_{1}\cdot D_{6}\cdot pS_{3} \right]}{dt}=q\left[ S_{1}\cdot D_{6}\cdot S_{3} \right]+k_{1a}^{+}\left[ S_{1} \right]\left[ D_{6}\cdot pS_{3} \right]$  $-k_{1a}^{-}\left[ S_{1}\cdot D_{6}\cdot pS_{3} \right]-q\left[ S_{1}\cdot D_{6}\cdot pS_{3} \right]-k_{3a}^{-}\left[ S_{1}\cdot D_{6}\cdot pS_{3} \right]$  $-\beta_{6}\left[ S_{1}\cdot D_{6}\cdot pS_{3} \right]-\gamma_{6}\left( \left[ pS_{1} \right]+\left[ pS_{3} \right] \right)\left[ S_{1}\cdot D_{6}\cdot pS_{3} \right]$ | (19) |
| $\frac{d\left[ pS_{1}\cdot D_{6}\cdot pS_{3} \right]}{dt}=q\left( \left[ S_{1}\cdot D_{6}\cdot pS_{3} \right]+\left[ pS_{1}\cdot D_{6}\cdot S_{3} \right] \right)$  $-\left[ pS_{1}\cdot D_{6}\cdot pS_{3} \right]\left( k_{1a}^{-}+k_{3a}^{-} \right)-\beta_{6}\left[ pS_{1}\cdot D_{6}\cdot pS_{3} \right]$  $-\gamma_{6}\left( \left[ pS_{1} \right]+\left[ pS_{3} \right] \right)\left[ pS_{1}\cdot D_{6}\cdot pS_{3} \right]$ | (20) |
| $\frac{d\left[ pS_{1} \right]}{dt}=k_{1a}^{-}\left( \left[ D_{6}\cdot pS_{1} \right]+\left[ S_{1}\cdot D_{6}\cdot pS_{1} \right]+\left[ S_{3}\cdot D_{6}\cdot pS_{1} \right]+\left[ pS_{3}\cdot D_{6}\cdot pS_{1} \right]+2\left[ pS_{1}\cdot D_{6}\cdot pS_{1} \right] \right)-d_{1}\left[ pS_{1} \right]$ | (21) |
| $\frac{d\left[ pS_{3} \right]}{dt}=k_{3a}^{-}\left( \left[ D_{6}\cdot pS_{3} \right]+\left[ S_{3}\cdot D_{6}\cdot pS_{3} \right]+\left[ S_{1}\cdot D_{6}\cdot pS_{3} \right]+\left[ pS_{1}\cdot D_{6}\cdot pS_{3} \right]+2\left[ pS_{3}\cdot D_{6}\cdot pS_{3} \right] \right)-d_{3}\left[ pS_{3} \right]$ | (22) |

Equations (23) – (55) are the ordinary differential equations (ODEs) describing the IL-27 signalling pathway, under the assumption of mass action kinetics.

| $\frac{d\left[ R_{1} \right]}{dt}=-r_{2,27}^{+}\left[ C_{2} \right]\left[ R_{1} \right]+r_{2,27}^{-}\left[ D_{27} \right]-\beta_{27}\left[ R_{1} \right]-\gamma_{27}\left( \left[ pS_{1} \right]+\left[ pS_{3} \right] \right)\left[ R_{1} \right]$ | (23) |
| --- | --- |
| $\frac{d\left[ R_{2} \right]}{dt}=-r_{1,27}^{+}\left[ R_{2} \right]\left[ L_{27} \right]+r_{1,27}^{-}\left[ C_{2} \right]-\beta_{27}\left[ R_{2} \right]-\gamma_{27}\left( \left[ pS_{1} \right]+\left[ pS_{3} \right] \right)\left[ R_{2} \right]$ | (24) |
| $\frac{d\left[ L_{27} \right]}{dt}=-r_{1,27}^{+}\left[ R_{2} \right]\left[ L_{27} \right]+r_{1,27}^{-}\left[ C_{2} \right]$ | (25) |
| $\frac{d\left[ C_{2} \right]}{dt}=r_{1,27}^{+}\left[ R_{2} \right]\left[ L_{27} \right]-r_{1,27}^{-}\left[ C_{2} \right]-r_{2,27}^{+}\left[ C_{2} \right]\left[ R_{1} \right]+r_{2,27}^{-}\left[ D_{27} \right]-\beta_{27}\left[ C_{2} \right]-\gamma_{27}\left( \left[ pS_{1} \right]+\left[ pS_{3} \right] \right)\left[ C_{2} \right]$ | (26) |
| $\frac{d\left[ D_{27} \right]}{dt}=r_{2,27}^{+}\left[ C_{2} \right]\left[ R_{1} \right]-r_{2,27}^{-}\left[ D_{27} \right]-\left( k_{1a}^{+}+k_{1b}^{+} \right)\left[ D_{27} \right]\left[ S_{1} \right]+k_{1a}^{-}\left( \left[ S_{1}\cdot D_{27} \right]+\left[ pS_{1}\cdot D_{27} \right] \right)+k_{1b}^{-}\left( \left[ D_{27}\cdot S_{1} \right]+\left[ D_{27}\cdot pS_{1} \right] \right)-\left( k_{3a}^{+}+k_{3b}^{+} \right)\left[ D_{27} \right]\left[ S_{3} \right]+k_{3a}^{-}\left( \left[ S_{3}\cdot D_{27} \right]+\left[ pS_{3}\cdot D_{27} \right] \right)+k_{3b}^{-}\left( \left[ D_{27}\cdot S_{3} \right]+\left[ D_{27}\cdot pS_{3} \right] \right)-\beta_{27}\left[ D_{27} \right]-\gamma_{27}\left( \left[ pS_{1} \right]+\left[ pS_{3} \right] \right)\left[ D_{27} \right]$ | (27) |
| $\frac{d\left[ S_{1} \right]}{dt}=-k_{1a}^{+}\left[ S_{1} \right]\left( \left[ D_{27} \right]+\left[ D_{27}\cdot S_{1} \right]+\left[ D_{27}\cdot pS_{1} \right]+\left[ D_{27}\cdot S_{3} \right]+\left[ D_{27}\cdot pS_{3} \right] \right)+k_{1a}^{-}\left( \left[ S_{1}\cdot D_{27} \right]+\left[ S_{1}\cdot D_{27}\cdot S_{1} \right]+\left[ S_{1}\cdot D_{27}\cdot pS_{1} \right]+\left[ S_{1}\cdot D_{27}\cdot S_{3} \right]+\left[ S_{1}\cdot D_{27}\cdot pS_{3} \right] \right)-k_{1b}^{+}\left[ S_{1} \right]\left( \left[ D_{27} \right]+\left[ S_{1}\cdot D_{27} \right]+\left[ pS_{1}\cdot D_{27} \right]+\left[ S_{3}\cdot D_{27} \right]+\left[ pS_{3}\cdot D_{27} \right] \right)+k_{1b}^{-}\left( \left[ D_{27}\cdot S_{1} \right]+\left[ S_{1}\cdot D_{27}\cdot S_{1} \right]+\left[ pS_{1}\cdot D_{27}\cdot S_{1} \right]+\left[ S_{3}\cdot D_{27}\cdot S_{1} \right]+\left[ pS_{3}\cdot D_{27}\cdot S_{1} \right] \right)+d_{1}\left[ pS_{1} \right]$ | (28) |
| $\frac{d\left[ S_{3} \right]}{dt}=-k_{3a}^{+}\left[ S_{3} \right]\left( \left[ D_{27} \right]+\left[ D_{27}\cdot S_{1} \right]+\left[ D_{27}\cdot pS_{1} \right]+\left[ D_{27}\cdot S_{3} \right]+\left[ D_{27}\cdot pS_{3} \right] \right)+k_{3a}^{-}\left( \left[ S_{3}\cdot D_{27} \right]+\left[ S_{3}\cdot D_{27}\cdot S_{1} \right]+\left[ S_{3}\cdot D_{27}\cdot pS_{1} \right]+\left[ S_{3}\cdot D_{27}\cdot S_{3} \right]+\left[ S_{3}\cdot D_{27}\cdot pS_{3} \right] \right)-k_{3b}^{+}\left[ S_{3} \right]\left( \left[ D_{27} \right]+\left[ S_{1}\cdot D_{27} \right]+\left[ pS_{1}\cdot D_{27} \right]+\left[ S_{3}\cdot D_{27} \right]+\left[ pS_{3}\cdot D_{27} \right] \right)+k_{3b}^{-}\left( \left[ D_{27}\cdot S_{3} \right]+\left[ S_{1}\cdot D_{27}\cdot S_{3} \right]+\left[ pS_{1}\cdot D_{27}\cdot S_{3} \right]+\left[ S_{3}\cdot D_{27}\cdot S_{3} \right]+\left[ pS_{3}\cdot D_{27}\cdot S_{3} \right] \right)+d_{3}\left[ pS_{3} \right]$ | (29) |
| $\frac{d\left[ S_{1}\cdot D_{27} \right]}{dt}=k_{1a}^{+}\left[ S_{1} \right]\left[ D_{27} \right]-k_{1a}^{-}\left[ S_{1}\cdot D_{27} \right]-q\left[ S_{1}\cdot D_{27} \right]-k_{1b}^{+}\left[ S_{1} \right]\left[ S_{1}\cdot D_{27} \right]+k_{1b}^{-}\left[ S_{1}\cdot D_{27}\cdot S_{1} \right]-k_{3b}^{+}\left[ S_{3} \right]\left[ S_{1}\cdot D_{27} \right]+k_{3b}^{-}\left[ S_{1}\cdot D_{27}\cdot S_{3} \right]+k_{1b}^{-}\left[ S_{1}\cdot D_{27}\cdot pS_{1} \right]+k_{3b}^{-}\left[ S_{1}\cdot D_{27}\cdot pS_{3} \right]-\beta_{27}\left[ S_{1}\cdot D_{27} \right]-\gamma_{27}\left( \left[ pS_{1} \right]+\left[ pS_{3} \right] \right)\left[ S_{1}\cdot D_{27} \right]$ | (30) |
| $\frac{d\left[ D_{27}\cdot S_{1} \right]}{dt}=k_{1b}^{+}\left[ S_{1} \right]\left[ D_{27} \right]-k_{1b}^{-}\left[ D_{27}\cdot S_{1} \right]-q\left[ D_{27}\cdot S_{1} \right]-k_{1a}^{+}\left[ S_{1} \right]\left[ D_{27}\cdot S_{1} \right]+k_{1a}^{-}\left[ S_{1}\cdot D_{27}\cdot S_{1} \right]-k_{3a}^{+}\left[ S_{3} \right]\left[ D_{27}\cdot S_{1} \right]+k_{3a}^{-}\left[ S_{3}\cdot D_{27}\cdot S_{1} \right]+k_{1a}^{-}\left[ pS_{1}\cdot D_{27}\cdot S_{1} \right]+k_{3a}^{-}\left[ pS_{3}\cdot D_{27}\cdot S_{1} \right]-\beta_{27}\left[ D_{27}\cdot S_{1} \right]-\gamma_{27}\left( \left[ pS_{1} \right]+\left[ pS_{3} \right] \right)\left[ D_{27}\cdot S_{1} \right]$ | (31) |
| $\frac{d\left[ S_{3}\cdot D_{27} \right]}{dt}=k_{3a}^{+}\left[ S_{3} \right]\left[ D_{27} \right]-k_{3a}^{-}\left[ S_{3}\cdot D_{27} \right]-q\left[ S_{3}\cdot D_{27} \right]-k_{3b}^{+}\left[ S_{3} \right]\left[ S_{3}\cdot D_{27} \right]+k_{3b}^{-}\left[ S_{3}\cdot D_{27}\cdot S_{3} \right]-k_{1b}^{+}\left[ S_{1} \right]\left[ S_{3}\cdot D_{27} \right]+k_{1b}^{-}\left[ S_{3}\cdot D_{27}\cdot S_{1} \right]+k_{3b}^{-}\left[ S_{3}\cdot D_{27}\cdot pS_{3} \right]+k_{1b}^{-}\left[ S_{3}\cdot D_{27}\cdot pS_{1} \right]-\beta_{27}\left[ S_{3}\cdot D_{27} \right]-\gamma_{27}\left( \left[ pS_{1} \right]+\left[ pS_{3} \right] \right)\left[ S_{3}\cdot D_{27} \right]$ | (32) |
| $\frac{d\left[ D_{27}\cdot S_{3} \right]}{dt}=k_{3b}^{+}\left[ S_{3} \right]\left[ D_{27} \right]-k_{3b}^{-}\left[ D_{27}\cdot S_{3} \right]-q\left[ D_{27}\cdot S_{3} \right]-k_{3a}^{+}\left[ S_{3} \right]\left[ D_{27}\cdot S_{3} \right]+k_{3a}^{-}\left[ S_{3}\cdot D_{27}\cdot S_{3} \right]-k_{1a}^{+}\left[ S_{1} \right]\left[ D_{27}\cdot S_{3} \right]+k_{1a}^{-}\left[ S_{1}\cdot D_{27}\cdot S_{3} \right]+k_{3a}^{-}\left[ pS_{3}\cdot D_{27}\cdot S_{3} \right]+k_{1a}^{-}\left[ pS_{1}\cdot D_{27}\cdot S_{3} \right]-\beta_{27}\left[ D_{27}\cdot S_{3} \right]-\gamma_{27}\left( \left[ pS_{1} \right]+\left[ pS_{3} \right] \right)\left[ D_{27}\cdot S_{3} \right]$ | (33) |
| $\frac{d\left[ pS_{1}\cdot D_{27} \right]}{dt}=-k_{1b}^{+}\left[ pS_{1}\cdot D_{27} \right]\left[ S_{1} \right]+k_{1b}^{-}\left[ pS_{1}\cdot D_{27}\cdot S_{1} \right]-k_{3b}^{+}\left[ pS_{1}\cdot D_{27} \right]\left[ S_{3} \right]+k_{3b}^{-}\left[ pS_{1}\cdot D_{27}\cdot S_{3} \right]+q\left[ S_{1}\cdot D_{27} \right]-k_{1a}^{-}\left[ pS_{1}\cdot D_{27} \right]+k_{1b}^{-}\left[ pS_{1}\cdot D_{27}\cdot pS_{1} \right]+k_{3b}^{-}\left[ pS_{1}\cdot D_{27}\cdot pS_{3} \right]-\beta_{27}\left[ pS_{1}\cdot D_{27} \right]-\gamma_{27}\left( \left[ pS_{1} \right]+\left[ pS_{3} \right] \right)\left[ pS_{1}\cdot D_{27} \right]$ | (34) |
| $\frac{d\left[ D_{27}\cdot pS_{1} \right]}{dt}=-k_{1a}^{+}\left[ D_{27}\cdot pS_{1} \right]\left[ S_{1} \right]+k_{1a}^{-}\left[ S_{1}\cdot D_{27}\cdot pS_{1} \right]-k_{3a}^{+}\left[ D_{27}\cdot pS_{1} \right]\left[ S_{3} \right]+k_{3a}^{-}\left[ S_{3}\cdot D_{27}\cdot pS_{1} \right]+q\left[ D_{27}\cdot S_{1} \right]-k_{1b}^{-}\left[ D_{27}\cdot pS_{1} \right]+k_{1a}^{-}\left[ pS_{1}\cdot D_{27}\cdot pS_{1} \right]+k_{3a}^{-}\left[ pS_{3}\cdot D_{27}\cdot pS_{1} \right]-\beta_{27}\left[ D_{27}\cdot pS_{1} \right]-\gamma_{27}\left( \left[ pS_{1} \right]+\left[ pS_{3} \right] \right)\left[ D_{27}\cdot pS_{1} \right]$ | (35) |
| $\frac{d\left[ pS_{3}\cdot D_{27} \right]}{dt}=-k_{3b}^{+}\left[ pS_{3}\cdot D_{27} \right]\left[ S_{3} \right]+k_{3b}^{-}\left[ pS_{3}\cdot D_{27}\cdot S_{3} \right]-k_{1b}^{+}\left[ pS_{3}\cdot D_{27} \right]\left[ S_{1} \right]+k_{1b}^{-}\left[ pS_{3}\cdot D_{27}\cdot S_{1} \right]+q\left[ S_{3}\cdot D_{27} \right]-k_{3a}^{-}\left[ pS_{3}\cdot D_{27} \right]+k_{3b}^{-}\left[ pS_{3}\cdot D_{27}\cdot pS_{3} \right]+k_{1b}^{-}\left[ pS_{3}\cdot D_{27}\cdot pS_{1} \right]-\beta_{27}\left[ pS_{3}\cdot D_{27} \right]-\gamma_{27}\left( \left[ pS_{1} \right]+\left[ pS_{3} \right] \right)\left[ pS_{3}\cdot D_{27} \right]$ | (36) |
| $\frac{d\left[ D_{27}\cdot pS_{3} \right]}{dt}=-k_{3a}^{+}\left[ D_{27}\cdot pS_{3} \right]\left[ S_{3} \right]+k_{3a}^{-}\left[ S_{3}\cdot D_{27}\cdot pS_{3} \right]-k_{1a}^{+}\left[ D_{27}\cdot pS_{3} \right]\left[ S_{1} \right]+k_{1a}^{-}\left[ S_{1}\cdot D_{27}\cdot pS_{3} \right]+q\left[ D_{27}\cdot S_{3} \right]-k_{3b}^{-}\left[ D_{27}\cdot pS_{3} \right]+k_{3a}^{-}\left[ pS_{3}\cdot D_{27}\cdot pS_{3} \right]+k_{1a}^{-}\left[ pS_{1}\cdot D_{27}\cdot pS_{3} \right]-\beta_{27}\left[ D_{27}\cdot pS_{3} \right]-\gamma_{27}\left( \left[ pS_{1} \right]+\left[ pS_{3} \right] \right)\left[ D_{27}\cdot pS_{3} \right]$ | (37) |
| $\frac{d\left[ S_{1}\cdot D_{27}\cdot S_{1} \right]}{dt}=k_{1a}^{+}\left[ S_{1} \right]\left[ D_{27}\cdot S_{1} \right]-k_{1a}^{-}\left[ S_{1}\cdot D_{27}\cdot S_{1} \right]$ $+k_{1b}^{+}\left[ S_{1}\cdot D_{27} \right]\left[ S_{1} \right]-k_{1b}^{-}\left[ S_{1}\cdot D_{27}\cdot S_{1} \right]-2q\left[ S_{1}\cdot D_{27}\cdot S_{1} \right]$  $-\beta_{27}\left[ S_{1}\cdot D_{27}\cdot S_{1} \right]-\gamma_{27}\left( \left[ pS_{1} \right]+\left[ pS_{3} \right] \right)\left[ S_{1}\cdot D_{27}\cdot S_{1} \right]$ | (38) |
| $\frac{d\left[ pS_{1}\cdot D_{27}\cdot S_{1} \right]}{dt}=k_{1b}^{+}\left[ pS_{1}\cdot D_{27} \right]\left[ S_{1} \right]-k_{1b}^{-}\left[ pS_{1}\cdot D_{27}\cdot S_{1} \right]$  $+q\left[ S_{1}\cdot D_{27}\cdot S_{1} \right]-q\left[ pS_{1}\cdot D_{27}\cdot S_{1} \right]-k_{1a}^{-}\left[ pS_{1}\cdot D_{27}\cdot S_{1} \right]$  $-\beta_{27}\left[ pS_{1}\cdot D_{27}\cdot S_{1} \right]-\gamma_{27}\left( \left[ pS_{1} \right]+\left[ pS_{3} \right] \right)\left[ pS_{1}\cdot D_{27}\cdot S_{1} \right]$ | (39) |
| $\frac{d\left[ S_{1}\cdot D_{27}\cdot pS_{1} \right]}{dt}=k_{1a}^{+}\left[ S_{1} \right]\left[ D_{27}\cdot pS_{1} \right]-k_{1a}^{-}\left[ S_{1}\cdot D_{27}\cdot pS_{1} \right]$  $+q\left[ S_{1}\cdot D_{27}\cdot S_{1} \right]-q\left[ S_{1}\cdot D_{27}\cdot pS_{1} \right]-k_{1b}^{-}\left[ S_{1}\cdot D_{27}\cdot pS_{1} \right]$  $-\beta_{27}\left[ S_{1}\cdot D_{27}\cdot pS_{1} \right]-\gamma_{27}\left( \left[ pS_{1} \right]+\left[ pS_{3} \right] \right)\left[ S_{1}\cdot D_{27}\cdot pS_{1} \right]$ | (40) |
| $\frac{d\left[ pS_{1}\cdot D_{27}\cdot pS_{1} \right]}{dt}=q\left( \left[ S_{1}\cdot D_{27}\cdot pS_{1} \right]+\left[ pS_{1}\cdot D_{27}\cdot S_{1} \right] \right)$  $-\left[ pS_{1}\cdot D_{27}\cdot pS_{1} \right]\left( k_{1a}^{-}+k_{1b}^{-} \right)-\beta_{27}\left[ pS_{1}\cdot D_{27}\cdot pS_{1} \right]$  $-\gamma_{27}\left( \left[ pS_{1} \right]+\left[ pS_{3} \right] \right)\left[ pS_{1}\cdot D_{27}\cdot pS_{1} \right]$ | (41) |
| $\frac{d\left[ S_{3}\cdot D_{27}\cdot S_{3} \right]}{dt}=k_{3a}^{+}\left[ S_{3} \right]\left[ D_{27}\cdot S_{3} \right]-k_{3a}^{-}\left[ S_{3}\cdot D_{27}\cdot S_{3} \right]$  $+k_{3b}^{+}\left[ S_{3}\cdot D_{27} \right]\left[ S_{3} \right]-k_{3b}^{-}\left[ S_{3}\cdot D_{27}\cdot S_{3} \right]-2q\left[ S_{3}\cdot D_{27}\cdot S_{3} \right]$  $-\beta_{27}\left[ S_{3}\cdot D_{27}\cdot S_{3} \right]-\gamma_{27}\left( \left[ pS_{1} \right]+\left[ pS_{3} \right] \right)\left[ S_{3}\cdot D_{27}\cdot S_{3} \right]$ | (42) |
| $\frac{d\left[ pS_{3}\cdot D_{27}\cdot S_{3} \right]}{dt}=k_{3b}^{+}\left[ pS_{3}\cdot D_{27} \right]\left[ S_{3} \right]-k_{3b}^{-}\left[ pS_{3}\cdot D_{27}\cdot S_{3} \right]$  $+q\left[ S_{3}\cdot D_{27}\cdot S_{3} \right]-q\left[ pS_{3}\cdot D_{27}\cdot S_{3} \right]-k_{3a}^{-}\left[ pS_{3}\cdot D_{27}\cdot S_{3} \right]$  $-\beta_{27}\left[ pS_{3}\cdot D_{27}\cdot S_{3} \right]-\gamma_{27}\left( \left[ pS_{1} \right]+\left[ pS_{3} \right] \right)\left[ pS_{3}\cdot D_{27}\cdot S_{3} \right]$ | (43) |
| $\frac{d\left[ S_{3}\cdot D_{27}\cdot pS_{3} \right]}{dt}=k_{3a}^{+}\left[ S_{3} \right]\left[ D_{27}\cdot pS_{3} \right]-k_{3a}^{-}\left[ S_{3}\cdot D_{27}\cdot pS_{3} \right]$  $+q\left[ S_{3}\cdot D_{27}\cdot S_{3} \right]-q\left[ S_{3}\cdot D_{27}\cdot pS_{3} \right]-k_{3b}^{-}\left[ S_{3}\cdot D_{27}\cdot pS_{3} \right]$  $-\beta_{27}\left[ S_{3}\cdot D_{27}\cdot pS_{3} \right]-\gamma_{27}\left( \left[ pS_{1} \right]+\left[ pS_{3} \right] \right)\left[ S_{3}\cdot D_{27}\cdot pS_{3} \right]$ | (44) |
| $\frac{d\left[ pS_{3}\cdot D_{27}\cdot pS_{3} \right]}{dt}=q\left( \left[ S_{3}\cdot D_{27}\cdot pS_{3} \right]+\left[ pS_{3}\cdot D_{27}\cdot S_{3} \right] \right)$  $-\left[ pS_{3}\cdot D_{27}\cdot pS_{3} \right]\left( k_{3a}^{-}+k_{3b}^{-} \right)-\beta_{27}\left[ pS_{3}\cdot D_{27}\cdot pS_{3} \right]$  $-\gamma_{27}\left( \left[ pS_{1} \right]+\left[ pS_{3} \right] \right)\left[ pS_{3}\cdot D_{27}\cdot pS_{3} \right]$ | (45) |
| $\frac{d\left[ S_{1}\cdot D_{27}\cdot S_{3} \right]}{dt}=k_{1a}^{+}\left[ S_{1} \right]\left[ D_{27}\cdot S_{3} \right]-k_{1a}^{-}\left[ S_{1}\cdot D_{27}\cdot S_{3} \right]$  $+k_{3b}^{+}\left[ S_{1}\cdot D_{27} \right]\left[ S_{3} \right]-k_{3b}^{-}\left[ S_{1}\cdot D_{27}\cdot S_{3} \right]-2q\left[ S_{1}\cdot D_{27}\cdot S_{3} \right]$  $-\beta_{27}\left[ S_{1}\cdot D_{27}\cdot S_{3} \right]-\gamma_{27}\left( \left[ pS_{1} \right]+\left[ pS_{3} \right] \right)\left[ S_{1}\cdot D_{27}\cdot S_{3} \right]$ | (46) |
| $\frac{d\left[ S_{3}\cdot D_{27}\cdot S_{1} \right]}{dt}=k_{3a}^{+}\left[ S_{3} \right]\left[ D_{27}\cdot S_{1} \right]-k_{3a}^{-}\left[ S_{3}\cdot D_{27}\cdot S_{1} \right]$  $+k_{1b}^{+}\left[ S_{3}\cdot D_{27} \right]\left[ S_{1} \right]-k_{1b}^{-}\left[ S_{3}\cdot D_{27}\cdot S_{1} \right]-2q\left[ S_{3}\cdot D_{27}\cdot S_{1} \right]$  $-\beta_{27}\left[ S_{3}\cdot D_{27}\cdot S_{1} \right]-\gamma_{27}\left( \left[ pS_{1} \right]+\left[ pS_{3} \right] \right)\left[ S_{3}\cdot D_{27}\cdot S_{1} \right]$ | (47) |
| $\frac{d\left[ pS_{1}\cdot D_{27}\cdot S_{3} \right]}{dt}=k_{3b}^{+}\left[ pS_{1}\cdot D_{27} \right]\left[ S_{3} \right]-k_{3b}^{-}\left[ pS_{1}\cdot D_{27}\cdot S_{3} \right]$  $+q\left[ S_{1}\cdot D_{27}\cdot S_{3} \right]-q\left[ pS_{1}\cdot D_{27}\cdot S_{3} \right]-k_{1a}^{-}\left[ pS_{1}\cdot D_{27}\cdot S_{3} \right]$  $-\beta_{27}\left[ pS_{1}\cdot D_{27}\cdot S_{3} \right]-\gamma_{27}\left( \left[ pS_{1} \right]+\left[ pS_{3} \right] \right)\left[ pS_{1}\cdot D_{27}\cdot S_{3} \right]$ | (48) |
| $\frac{d\left[ pS_{3}\cdot D_{27}\cdot S_{1} \right]}{dt}=k_{1b}^{+}\left[ pS_{3}\cdot D_{27} \right]\left[ S_{1} \right]-k_{1b}^{-}\left[ pS_{3}\cdot D_{27}\cdot S_{1} \right]$  $+q\left[ S_{3}\cdot D_{27}\cdot S_{1} \right]-q\left[ pS_{3}\cdot D_{27}\cdot S_{1} \right]-k_{3a}^{-}\left[ pS_{3}\cdot D_{27}\cdot S_{1} \right]$  $-\beta_{27}\left[ pS_{3}\cdot D_{27}\cdot S_{1} \right]-\gamma_{27}\left( \left[ pS_{1} \right]+\left[ pS_{3} \right] \right)\left[ pS_{3}\cdot D_{27}\cdot S_{1} \right]$ | (49) |
| $\frac{d\left[ S_{1}\cdot D_{27}\cdot pS_{3} \right]}{dt}=k_{1a}^{+}\left[ S_{1} \right]\left[ D_{27}\cdot pS_{3} \right]-k_{1a}^{-}\left[ S_{1}\cdot D_{27}\cdot pS_{3} \right]$  $+q\left[ S_{1}\cdot D_{27}\cdot S_{3} \right]-q\left[ S_{1}\cdot D_{27}\cdot pS_{3} \right]-k_{3b}^{-}\left[ S_{1}\cdot D_{27}\cdot pS_{3} \right]$  $-\beta_{27}\left[ S_{1}\cdot D_{27}\cdot pS_{3} \right]-\gamma_{27}\left( \left[ pS_{1} \right]+\left[ pS_{3} \right] \right)\left[ S_{1}\cdot D_{27}\cdot pS_{3} \right]$ | (50) |
| $\frac{d\left[ S_{3}\cdot D_{27}\cdot pS_{1} \right]}{dt}=k_{3a}^{+}\left[ S_{3} \right]\left[ D_{27}\cdot pS_{1} \right]-k_{3a}^{-}\left[ S_{3}\cdot D_{27}\cdot pS_{1} \right]$  $+q\left[ S_{3}\cdot D_{27}\cdot S_{1} \right]-q\left[ S_{3}\cdot D_{27}\cdot pS_{1} \right]-k_{1b}^{-}\left[ S_{3}\cdot D_{27}\cdot pS_{1} \right]$  $-\beta_{27}\left[ S_{3}\cdot D_{27}\cdot pS_{1} \right]-\gamma_{27}\left( \left[ pS_{1} \right]+\left[ pS_{3} \right] \right)\left[ S_{3}\cdot D_{27}\cdot pS_{1} \right]$ | (51) |
| $\frac{d\left[ pS_{1}\cdot D_{27}\cdot pS_{3} \right]}{dt}=q\left( \left[ S_{1}\cdot D_{27}\cdot pS_{3} \right]+\left[ pS_{1}\cdot D_{27}\cdot S_{3} \right] \right)$  $-\left[ pS_{1}\cdot D_{27}\cdot pS_{3} \right]\left( k_{1a}^{-}+k_{3b}^{-} \right)-\beta_{27}\left[ pS_{1}\cdot D_{27}\cdot pS_{3} \right]$  $-\gamma_{27}\left( \left[ pS_{1} \right]+\left[ pS_{3} \right] \right)\left[ pS_{1}\cdot D_{27}\cdot pS_{3} \right]$ | (52) |
| $\frac{d\left[ pS_{3}\cdot D_{27}\cdot pS_{1} \right]}{dt}=q\left( \left[ S_{3}\cdot D_{27}\cdot pS_{1} \right]+\left[ pS_{3}\cdot D_{27}\cdot S_{1} \right] \right)$  $-\left[ pS_{3}\cdot D_{27}\cdot pS_{1} \right]\left( k_{3a}^{-}+k_{1b}^{-} \right)-\beta_{27}\left[ pS_{3}\cdot D_{27}\cdot pS_{1} \right]$  $-\gamma_{27}\left( \left[ pS_{1} \right]+\left[ pS_{3} \right] \right)\left[ pS_{3}\cdot D_{27}\cdot pS_{1} \right]$ | (53) |
| $\frac{d\left[ pS_{1} \right]}{dt}=k_{1a}^{-}\left( \left[ pS_{1}\cdot D_{27} \right]+\left[ pS_{1}\cdot D_{27}\cdot S_{1} \right]+\left[ pS_{1}\cdot D_{27}\cdot pS_{1} \right]+\left[ pS_{1}\cdot D_{27}\cdot S_{3} \right]+\left[ pS_{1}\cdot D_{27}\cdot pS_{3} \right] \right)+k_{1b}^{-}\left( \left[ D_{27}\cdot pS_{1} \right]+\left[ S_{1}\cdot D_{27}\cdot pS_{1} \right]+\left[ pS_{1}\cdot D_{27}\cdot pS_{1} \right]+\left[ S_{3}\cdot D_{27}\cdot pS_{1} \right]+\left[ pS_{3}\cdot D_{27}\cdot pS_{1} \right] \right)-d_{1}\left[ pS_{1} \right]$ | (54) |
| $\frac{d\left[ pS_{3} \right]}{dt}=k_{3a}^{-}\left( \left[ pS_{3}\cdot D_{27} \right]+\left[ pS_{3}\cdot D_{27}\cdot S_{3} \right]+\left[ pS_{3}\cdot D_{27}\cdot pS_{3} \right]+\left[ pS_{3}\cdot D_{27}\cdot S_{1} \right]+\left[ pS_{3}\cdot D_{27}\cdot pS_{1} \right] \right)+k_{3b}^{-}\left( \left[ D_{27}\cdot pS_{3} \right]+\left[ S_{3}\cdot D_{27}\cdot pS_{3} \right]+\left[ pS_{3}\cdot D_{27}\cdot pS_{3} \right]+\left[ S_{1}\cdot D_{27}\cdot pS_{3} \right]+\left[ pS_{1}\cdot D_{27}\cdot pS_{3} \right] \right)-d_{3}\left[ pS_{3} \right]$ | (55) |
